# Cov^2^MS: An Automated and Quantitative Matrix-Independent Assay for Mass Spectrometric Measurement of SARS-CoV-2 Nucleocapsid Protein

**DOI:** 10.1021/acs.analchem.2c01610

**Published:** 2022-12-09

**Authors:** Bart Van Puyvelde, Katleen Van Uytfanghe, Laurence Van Oudenhove, Ralf Gabriels, Tessa Van Royen, Arne Matthys, Morteza Razavi, Richard Yip, Terry Pearson, Nicolas Drouin, Jan Claereboudt, Dominic Foley, Robert Wardle, Kevin Wyndham, Thomas Hankemeier, Donald Jones, Xavier Saelens, Geert Martens, Christophe P. Stove, Dieter Deforce, Lennart Martens, Johannes P.C. Vissers, N. Leigh Anderson, Maarten Dhaenens

**Affiliations:** † ProGenTomics, Laboratory of Pharmaceutical Biotechnology, Ghent University, 9000 Ghent, Belgium; ‡ Laboratory of Toxicology, Department of Bioanalysis, Faculty of Pharmaceutical Sciences, Ghent University, 9000 Ghent, Belgium; § Waters Corporation, 2600 Antwerp, Belgium; ∥ VIB-UGent Center for Medical Biotechnology, VIB, 9000 Ghent, Belgium; ⊥ Department of Biomolecular Medicine, Ghent University, 9000 Ghent, Belgium; # Department of Biochemistry and Microbiology, Ghent University, Ghent 9000 Belgium; ∇ SISCAPA Assay Technologies, Inc., Box 53309, Washington, DC 20009, United States; □ Department of Biochemistry and Microbiology, University of Victoria, Victoria, BC V8P 5C2, Canada; ○ Division of Systems Biomedicine and Pharmacology, Leiden Academic Centre for Drug Research, Leiden University, 2333 AL Leiden, The Netherlands; ◆ Waters Corporation, Wilmslow SK9 4AX, United Kingdom; △ Waters Corporation, Milford, Massachusetts 01757, United States; ¶ Leicester Cancer Research Centre, RKCSB, Cardiovascular Research Centre, Glenfield Hospital, University of Leicester, Leicester LE1 7RH, United Kingdom; ▷ John and Lucille van Geest Biomarker Facility, Leicester LE3 9QP, United Kingdom; & The Department of Chemical Pathology and Metabolic Diseases, Leicester Royal Infirmary, Level 4, Sandringham Building, Leicester LE1 7RH, United Kingdom; ● AZ Delta Medical Laboratories, AZ Delta General Hospital, 8800 Roeselare, Belgium

## Abstract

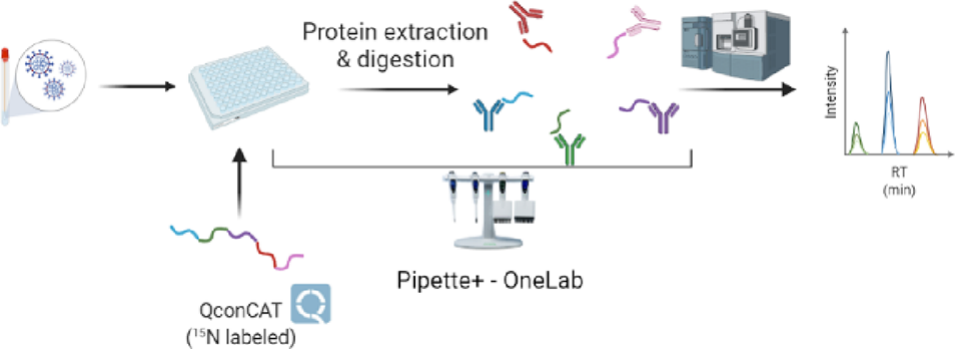

The pandemic readiness toolbox needs to be extended, targeting different biomolecules, using orthogonal experimental set-ups. Here, we build on our Cov-MS effort using LC–MS, adding SISCAPA technology to enrich proteotypic peptides of the SARS-CoV-2 nucleocapsid (N) protein from trypsin-digested patient samples. The Cov^2^MS assay is compatible with most matrices including nasopharyngeal swabs, saliva, and plasma and has increased sensitivity into the attomole range, a 1000-fold improvement compared to direct detection in a matrix. A strong positive correlation was observed with qPCR detection beyond a quantification cycle of 30–31, the level where no live virus can be cultured. The automatable sample preparation and reduced LC dependency allow analysis of up to 500 samples per day per instrument. Importantly, peptide enrichment allows detection of the N protein in pooled samples without sensitivity loss. Easily multiplexed, we detect variants and propose targets for Influenza A and B detection. Thus, the Cov^2^MS assay can be adapted to test for many different pathogens in pooled samples, providing longitudinal epidemiological monitoring of large numbers of pathogens within a population as an early warning system.

## Introduction

The COVID-19 pandemic starkly revealed that humanity is ill-prepared for such global catastrophes. Rising population density, increasing interactions between people and animals in wild habitats, and global mobility make humanity increasingly prone to emergent large-scale infections, i.e., future pandemics. It is clear that pandemic readiness needs to be extended, providing tools that allow early warning of threatening pathogens. In particular, robust highly sensitive and specific diagnostics allow screening of large populations for monitoring of pathogen load, disease progression, and treatment efficacy, perhaps allowing triage of patients when resources are scarce. Although not without their problems, current nucleic acid amplification tests such as reverse transcription-quantitative polymerase chain reaction (RT-qPCR) have been, and will likely remain, a major tool for large-scale screening. These types of tests work by detecting amplified levels of pathogen-derived nucleic acid and are excellent for testing for exposure to the pathogen. However, there is a need for measuring viral load as a more direct determination of productive virus infection for monitoring disease progression and treatment to complement the notoriously sensitive PCR tests. Indeed, the most recent estimation for life virus and thus infectivity in patients was below qPCR cycle thresholds Ct 31 on the E-gene, effectively showing that higher detections are questionable assets to population testing.^1^

Several groups have suggested that liquid chromatography coupled to mass spectrometry (LC–MS) might be a method of choice for unequivocal detection of SARS-CoV-2 proteins (Supplementary Figure S1A).^2−12^ In phase 1 of a community-based effort involving 15 labs and industrial partners, named Cov-MS, we examined the current state of the art for direct LC–MS detection of viral proteins in the most commonly used virus transport media.^2^ We noticed that LC–MS assays for several of the structural SARS-CoV-2 proteins can be developed without having to change sample matrices or standard procedures. Importantly, the different reports on the use of MS essentially detected many of the same SARS-CoV-2 biomarker peptides, irrespective of the model of their LC–MS instruments, the sample preparation platform, or methods used for bioinformatics analysis. In other words, preferentially detected peptides in a preliminary screen turn out to be universally applicable. Thus, a phase 1 assay can be developed quickly and without the requirement for clinicians to adopt different sampling procedures.

However, there are inherent shortcomings in standard LC–MS methods for peptide identification and quantification. While the latest generation instruments generate enough peptide multiple reaction monitoring (MRM) signal for clinical relevance, the limiting factor is the signal-to-noise ratio.^13^ It is predominantly the presence of a matrix that interferes with and hampers robustness and sensitivity. In addition to interferences in the matrices, contaminating and potentially interfering protein molecules are present in most common viral universal transport media. These contaminate the instrument, limit the amount of sample that can be analyzed (on-column limitations), suppress ionization of analyte peptides, require long chromatography times for peptide separation, and hamper data interpretation.

Here, we build on previous insights and describe the development of a second-generation assay, which we named Cov^2^MS, by implementing SISCAPA immuno-MS peptide quantitation technology to eliminate interferences and to reduce liquid chromatography time, allowing higher throughput. As shown earlier on patient samples, SISCAPA peptide enrichment technology allows very sensitive SARS-CoV-2 protein detection corresponding to Ct equivalents ranging from 21 to 34 yet with much higher quantitative precision.^14,15^ Here, we report on the full impact of this sample preparation step that enables a more generalized diagnostic method that could readily be deployed in clinical laboratories. We illustrate that the use of peptide immuno-enrichment technology for MS-based SARS-CoV-2 detection essentially addresses the most important issues identified in phase 1 of the Cov-MS assay. The Cov^2^MS assay can now be applied for the analysis of samples in almost any matrix, i.e., transport medium or biological background, with limited compromise while increasing its sensitivity into the attomole range, enabling a strong positive correlation with RT-qPCR-based viral RNA levels at least up to Ct 30. The entire workflow is amenable to automation using commercially available liquid handling robots. A single robot can process up to 500 samples in an 8 h shift, and processing 500 samples per day per instrument is feasible with a cycle time of approximately 2 min as presented in this manuscript. Importantly, we have demonstrated that using SISCAPA enrichment, the specimen from one positive patient can be pooled with samples from at least 30 negative patients without noticeable loss in sensitivity. An exciting observation was that during the development of this updated Cov^2^MS assay, two SARS-CoV-2 variants emerged that spread in the population, including the notorious Delta B.1.617.2 variant-of-concern (VoC). Both variants have mutations in the peptides used in the assay. The mutated forms of the peptides were both enriched using the SISCAPA protocol and were identified by MS, indicating that the Cov^2^MS assay can differentiate between the Delta and other variants simultaneously.

As a future perspective, we propose to establish a candidate target panel of SISCAPA-based LC–MS assays, which will be able to detect peptides from Influenza A and B viruses at similar or improved sensitivity as SARS-CoV-2. We propose the use of infection proteomics as a general term for future extensions of the assay. Indeed, next-generation tests will have the potential to detect several pathogens simultaneously in almost all media at sensitivities matching infectivity limits with considerably higher quantitative accuracies and unequivocal identification of the analyte detected. Especially in light of pandemic readiness, we foresee longitudinal population-wide monitoring of up to a dozen respiratory viruses in pooled patient samples as an early warning system for impending epidemics and pandemics.^13^

## Materials and Methods

### Recombinant Proteins and Automation

Recombinant nucleoprotein (N) of SARS-CoV-2 (2019-nCov), Influenza A (A/Wisconsin/588/2019–A/Victoria/2570/2019), and Influenza B (B/Phuket/3073/2013) was produced in insect cells with a baculovirus expression system (Sino Biological, Beijing, China). The SARS-CoV-2 sample preparation protocol was automated using an Andrew Alliance Pipette+ and Shaker+ connected device (Waters Corporation, Milford, MA, USA) both operated via the OneLab platform.

### Samples

Residual Covid-19 nasopharyngeal patient samples were obtained from the AZ Delta Hospital, Roeselare, Belgium, with approval of the University Hospital Ghent ethics committee (BC-09263). These samples were analyzed at the clinical laboratory of the AZ Delta Hospital using the Allplex 2019-nCoV RT-PCR assay from Seegene Inc.^16^

Lyophilized recombinant N protein was reconstituted to a concentration of 0.1 μg/μL in 100 mM NH_4_HCO_3_. A 50 fmol/μL calibration standard of N was prepared in SARS-CoV-2-negative nasopharyngeal swab pools of different media, i.e., 100 mM NH_4_HCO_3_, Copan Universal Transport Medium (UTM), Bioer UTM, Sigma Virocult, eSwab, PBS, plasma, synthetic saliva (saliva substitute, donated by the University of Leicester), and patient saliva. A serial dilution in SARS-CoV-2-negative nasopharyngeal swab pools was made: 10000, 2000, 400, 80, 16, 4, 2, and 0 amol/μL. An equimolar dilution series of recombinant N protein from Influenza A (Victoria/2570/2019), Influenza B (Phuket/3073/2013), and SARS-CoV-2 (root (L) strain) was generated in 100 mM NH_4_HCO_3_.

### Protein Extraction and Digestion

Each sample was prepared using the same workflow, namely proteins in 180 μL of undiluted sample (60 μL for plasma) were precipitated by adding seven volumes of ice-cold acetone (−20 °C). After centrifugation at 16,000*g*, at 0 ° C, the supernatant was discarded and 1 μg of trypsin/Lys-C mix (Promega, Madison, WI, USA) in 150 μL of 100 mM NH_4_HCO_3_ was added. Prior to incubation for 30 min at 37 °C, the samples were transferred from Protein LoBind tubes into a 96-well sample collection plate (Waters Corporation). To inhibit further digestion, 50 μL of a 0.22 mg/mL TLCK (Sigma-Aldrich, St. Louis, MO, USA) in 10 mM HCl solution was added to each sample followed by mixing the plate on the Shaker+ at 1000 rpm for 5 min at room temperature. Each sample was spiked with 100 fmol of the Cov-MS QconCAT standard (Polyquant, Bad Abbach, Germany) before acetone treatment to precipitate proteins.^17^

### Peptide Selection

Proteotypic SARS-CoV-2 peptides were selected and validated in the Cov-MS consortium.^2^ Complemented with the literature, this allowed us to select the “best” peptides (Figure S1A) as surrogates for the viral proteins. The N protein encoding gene has exhibited fewer mutations than other SARS-CoV-2 structural proteins^18^ and is also the most abundant of the viral structural proteins.^3,19,20^

### Anti-peptide Antibodies and Magnetic Bead Immunoadsorbents

Affinity-purified anti-peptide polyclonal rabbit antibodies specific for 10 different peptides of N protein were prepared and tested in SISCAPA peptide enrichment-MS assays. Of these, six polyclonal triggered the derivation of rabbit anti-peptide monoclonal antibodies (RabMAbs) from the same rabbits. The RabmAbs were screened by proprietary methods to allow selection of highly specific, high-affinity anti-peptide antibodies capable of binding low-abundance peptides from solution and retaining them through extensive washing steps designed to minimize non-specific background. The selected antibodies were produced as recombinant proteins, and all have sub-nanomolar affinities and slow off rates. Antibodies specific for six peptides (ADETQALPQR, AYNVTQAFGR, DGIIWVATEGALNTPK, NPANNAAIVLQLPQGTTLPK, GQGVPINTNSSPDDQIGYYR, and KQQTVTLLPAAD-LDDFSK) were covalently coupled using dimethyl pimelimidate to protein G magnetic beads (Life Technologies) in 1× PBS with 0.03% CHAPS and stored at 4–8 ° C.

### Peptide Enrichment

Antibody-coupled magnetic bead immunoadsorbents were resuspended fully by vortex mixing. Equal volumes of the six bead suspensions were mixed, and 60 μL of the mixture was added to the trypsin-digested samples once tryptic digestion activity had been neutralized. Plates were put on the Shaker+ and first shaken at 1400 rpm for 3 min prior to a 1 h incubation at 1100 rpm at room temperature. After incubation, the plates were placed on a custom-made magnet array (SISCAPA Assay Technologies) for 1 min and once the beads had been drawn to the sides of each well, the supernatant (approximately 260 μL) was removed. The beads were then washed by addition of 150 μL of wash buffer (0.03% CHAPS, 1× PBS) to each sample followed by resuspending the beads by shaking the plates at 1400 rpm for 1 min. The sample plates were then again placed on the magnet array, and the supernatant was removed. The washing step was performed a second time. Subsequently, the beads were resuspended in 50 μL of elution buffer (1% formic acid, 0.03% CHAPS) and mixed at 1400 rpm for 5 min at room temperature. Finally, after placing the plates on the magnetic plate, the eluents containing the eluted peptides were transferred to a QuanRecovery 96-well plate (Waters Corporation) for LC–MS analysis.

### LC–MS Detection and Quantification

LC separation was performed on an ACQUITY UPLC I-Class FTN system, with a Binary Solvent Manager with column selection valves (Waters Corporation). Ten microliters of the enriched sample was injected onto an ACQUITY Premier Peptide BEH C18 column (2.1 mm × 30 (or 50) mm, 1.7 μm, 300 Å) column (Waters Corporation). Peptide separation was performed using a gradient elution of mobile phase A containing LC–MS-grade deionized water with 0.1% (v/v) formic acid and mobile phase B containing LC–MS-grade acetonitrile with 0.1% (v/v) formic acid. A Xevo TQ-XS tandem MS (Waters Corporation, Wilmslow, UK), operating in positive electrospray ionization, was used for the detection and quantification of the peptides. Details on the LC gradients and MS parameters can be retrieved from Supplementary Methods and Table S1.

Skyline (version 21.1) was used to process the raw LC–MS data using a template file containing the six target peptides. Peak integration boundaries were automatically set on the heavy standard and manually reviewed before exporting a report containing the peptide-modified sequence, transition, area, and height among others.

The mass spectrometry MRM and DIA-MS proteomics data have been deposited to the ProteomeXchange Consortium via the Panorama Public partner repository with the dataset identifier PXD031401.^21,22^

## Results and Discussion

### Development and Preliminary Validation of SISCAPA Anti-peptide Antibodies

Throughout the SARS-CoV-2 pandemic, clinical laboratories have switched between providers of nasopharyngeal swabs and transport media because of fluctuations in the supply chain. All of these were validated for RT-qPCR compatibility. However, we have shown that transport media can heavily impact the detectability of viral proteins using MS.^2^ Therefore, to remove interferences as much as possible, SISCAPA-compatible high-affinity anti-peptide antibodies were produced against a selection of proteotypic, surrogate N protein peptides (Figure S1A, red arrowheads). These peptides were originally also incorporated into the Cov-MS QconCAT heavy internal standard (PolyQuant, Bad Abbach, Germany), which was also used here throughout the assay development process.^17^

**Figure 1:**
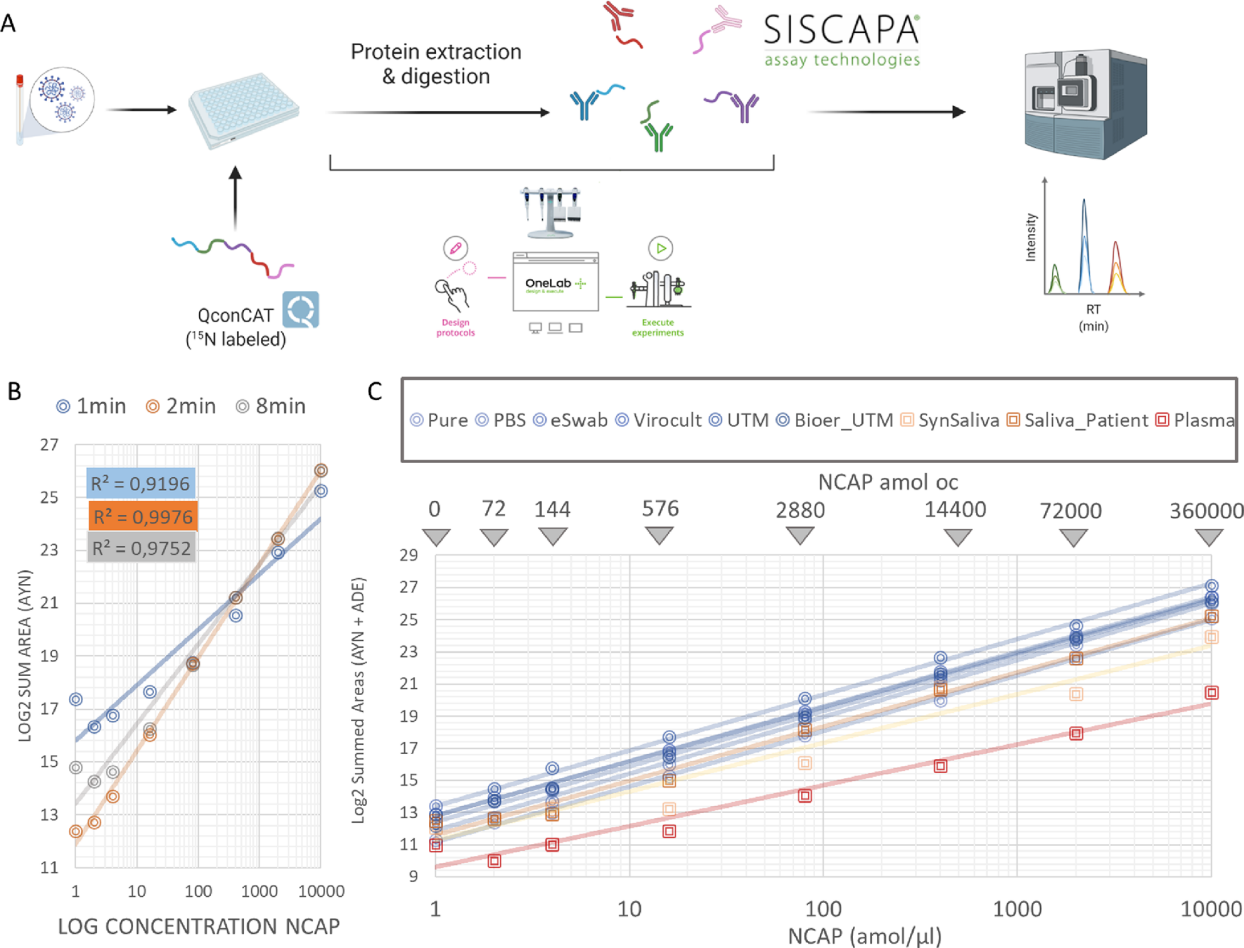
Validation of the peptide enrichment protocol using SISCAPA technology. (A) Schematic representation of the SISCAPA workflow. **(**B) Comparison of different gradient lengths and their linearity for detecting the AYN peptide. (C) Linearity of response of the dilution series in different matrices. The amount that is loaded on the column (oc) is indicated at the top. This is the amount of peptide following enrichment (calculation described in Supplementary Methods).

An in-house comparison of the performance of polyclonal and monoclonal antibodies for two of the peptides is shown in Figure S1B. The increased performance of monoclonal antibodies is especially pronounced at lower concentrations, effectively increasing the sensitivity of the assay where it is most needed. The “addition-only” SISCAPA protocol relies on the use of magnetic bead immunoadsorbents for target peptide purification and can therefore be automated to both increase the sample throughput and to reduce technical variation, i.e., increase quantitative accuracy. Therefore, we optimized a protocol for the digestion and magnetic bead purification using the programmable Pipette+ system (Figure 1A) and later transferred to the Andrew+ liquid handling robot for determining precision, recovery, and analytical sensitivity.

After removal of matrix molecules by the peptide enrichment method, the LC gradient can be shortened, narrowing the chromatographic peaks and providing increased detectability without the risk of increasing interferences. We compared the original Cov-MS gradient of 8 min with a gradient of 2 min for all peptides and a 1 min gradient for only two peptides (AYN and KQQ) and assessed the linearity of a dilution series in PBS matrix (Figure S2). Overall, a 2 min gradient demonstrated the highest linearity, with the ADE and AYN peptides performing the best in terms of MRM sensitivity, especially in the low-intensity range close to the lower limit of quantification. Figure 1B shows the response for AYN in log2 transformed intensity (Log2Int). Notably, using a 2 min gradient, a total of 500 patients per instrument per day could potentially be analyzed. While such patient sample batch sizes are not yet available, 600 samples were run in less than 96 h on two separate occasions during the work reported here, including dilution series comprising a total of nine different matrices. No decline in instrument performance was apparent. In summary, a 2 min gradient provided the highest linearity, detectability, and throughput and was selected for subsequent analyses.

### Analytical Performance of the Method

Two peptides (ADE and AYN) were selected for further work based on their stability and linearity on a 2 min gradient in all the transport media tested (Figure S3). The analytical performance of the method was further assessed on AYN and ADE synthetic peptide spikes prepared using the Andrew+ liquid handling robot (Andrew Alliance). The functional sensitivity, expressed as limit of quantitation, was 3 amol/μL, where the intra- and inter-day %CV and bias were still <20% (Figure S4). Note that the integration of the raw MS signal is software-dependent and can greatly impact the limit of detection, which is therefore not explicitly calculated here but is expressed as a function of the RT-qPCR Ct value later.

### Mitigating Matrix Effects

Enabling direct peptide detection in a variety of transport and biological matrices significantly broadens the applicability of the method. Therefore, we assessed the linearity of response for six N protein peptides in a dilution series in (i) six different (viral transport) media and (ii) two different biological matrices, saliva and plasma, as well as a synthetic surrogate for saliva. Figure S4 shows that the %CV of three different preparations in these different media increases with decreasing signal intensity for all transport media, as expected for MS measurements.^23^ To illustrate the transferability of the method, a similar dilution series in a Bioer UTM was measured on a SCIEX Triple Quad 7500 System, with very comparable %CV and linearity (Figure S3, inset). Notably, for the combined sum of intensities extracted from the open-source Skyline freeware for these two peptides, the blank signal is lower than that at 72 amol on-column in all transport media. Previously, a theoretical detection limit of 40 amol was proposed by us in samples without matrix, based on the extrapolation of the signal detection for pure N protein preparations.^2^ This in turn demonstrates the efficiency of the enrichment strategy. In fact, for the UTM, this dilution series implies at least a 100-fold more sensitive detection compared to the phase 1 Cov-MS, wherein the detection limit estimation was heavily compromised by interferences.^2^ Notably, the biological matrices and synthetic saliva show a larger variation in measurement. Still, the signal intensity (area sum of the MRM transitions) in all media is strongly linear, suggesting that all can be analyzed by the Cov^2^MS assay (Figure 1C).^24,25^ The detection of viral peptides in plasma creates the possibility for direct detection of viral load in blood, in turn enabling the assessment of disease status, clinical prognostic value, and treatment monitoring. It will be interesting to test its utility for monitoring long COVID, perhaps using additional biomarkers for inflammation or immune responses in a multiplexed analysis.^26^

### Comparison between RT-qPCR and SISCAPA-LC–MS Performed on Patient Samples

**Figure 2:**
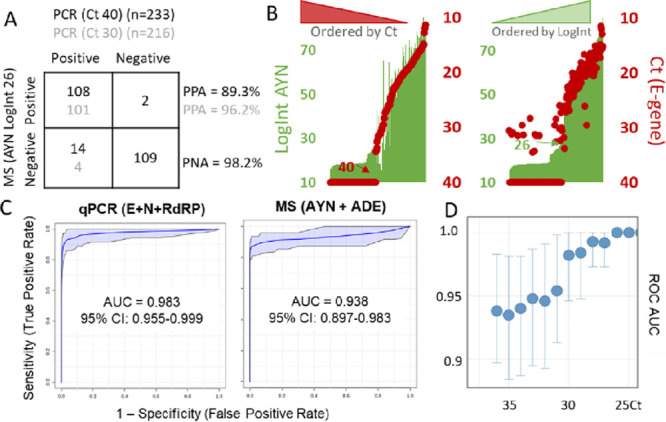
Comparison between RT-qPCR and SISCAPA-LC–MS performed on 233 patient samples in three different transport media. (A) A patient sample batch in different media displays a high percent positive (PPA = TP/(TP + FN)) and negative agreement (PNA = TN/(TN + FP)) between RT-qPCR (Ct) and MS (LogInt), especially below Ct 30 (gray numbers). (B) Secondary axis plots of the raw measurements of E-gene Ct (red dots) and AYN logarithmically transformed MS intensities (LogInt) (green bars) for patients sorted from high to low Ct (left) and low to high LogInt (right). A strong linear correlation illustrates the level of agreement between both tests. The patient samples were only prepared once since we only had access to the residual volume (<300 μL) after clinical RT-qPCR analysis. (C) ROC with true positives defined by either RT-qPCR (left) or MS (right). AUC: area under the curve. (D) ROC AUCs for each Ct value separately. Up to Ct 26, there is perfect agreement (AUC = 1). Above Ct 30, a noticeable drop-off to an AUC of 0.95 can be seen, suggesting that from here on, both diagnostic tests start to disagree. Error bars indicate the 95% confidence interval (CI).

While five monoclonal antibody reagents were used on a patient cohort of 233 samples, we first assessed the performance of the AYN peptide as suggested by Hober et al.^14^ A 2 min gradient was used, and three different viral transport media were included, i.e., PBS, Bioer UTM, and Bioer VIM (Viral Inactivation Medium). Note that in order to define the sensitivity and specificity for MS analysis, high patient numbers and a ground truth are required, e.g., by using true negative patients sampled before the pandemic. Such samples were unavailable to us. Therefore, a binary comparison to RT-qPCR (positive and negative) should rather be expressed in percent positive agreement (PPA) and percent negative agreement (PNA).^14,27^Figure 2A depicts these numbers when an initial summed MRM intensity of AYN of 26 LogInt is used to define positive patients for MS, and <Ct 40 is used for the RT-qPCR threshold (122 RT-qPCR-positive and 111 RT-qPCR-negative patients). As suggested by Hober et al., we also plotted these numbers against an RT-qPCR positivity threshold of Ct 30, leaving out the patients between Ct 30 and 40. This clearly illustrates how well RT-qPCR and MS agree, especially up to Ct 30, with 96.2% PPA and 98.2% NPA, respectively.^14^

However, there are several concerns with this representation. First, both tests report a continuous measure rather than a binary outcome and a threshold needs to be chosen to define “positive” and “negative”, arguably not trivial for either test. In fact, the positivity threshold for RT-qPCR varies greatly between assays and should probably be set to Ct 31 in light of recent insights on infectivity.^1^ Second, the numbers reported in the matrix are a direct function of the Ct distribution in the patient population tested. Figure S5A illustrates that shifting, e.g., the positivity threshold of qPCR from Ct 40 to Ct 35 would not impact PPA or PNA because there are no patient samples in that region. Third, RT-qPCR is not well suited to define the amount of RNA in copies/mL, with measurements sometimes differing >1000-fold between laboratories.^28^ MS on the other hand is considered a quantitative and accurate analytical tool, an asset typically not attributed to other protein detection technologies, such as lateral flow antigen tests.^29^ In other words, the Ct reported for a patient can vary greatly and thus defining RT-qPCR as the ground truth or golden standard does not objectify the comparison. Finally, there is a potential underlying biological reason why RNA and protein do not completely correlate, i.e., the stage of infection.^30,31^ Indeed, while RNA and protein levels will most probably rise in parallel at the onset of infection, it is known that residual RNA can still be detected over a month following infection when the disease symptoms are no longer apparent.^1,31,32^

Therefore, the raw results from both tests are first depicted on two secondary axis plots (Figure 2B). Each patient (*x* axis) is represented by its two respective measurements, i.e., LogInt of the AYN peptide (green bars, left axis) and the Ct value for the E-gene (red dots, right axis). As reported earlier, the LogInt of positive patients strongly correlates linearly (*R*^2^ = 0.86) to the Ct value, which is an exponential metric depicting the number of doublings required for detection.^2,14^ In both plots, patients were sorted from low to high virus measurement, i.e., from high to low Ct and from low to high LogInt**.** While only two patients with Ct 40 had a LogInt for AYN > 26, evenly spread patients have Ct values of 28–35 below this MS intensity threshold, all the way down to the patient with the lowest value as measured by MS. These samples are either false positives by RT-qPCR or false negatives by MS as depicted in Figure 2A. Alternatively, this raises the possibility that these patients were in an early or late stage of infection.^31^

Figure 2C shows the receiver operating curves (ROC) with RT-qPCR and MS respectively defining the “ground truth” at the clinical diagnosis level of Ct >36 or the summed LogInt for the AYN + ADE peptides at 26.6, which was inferred from the median and median absolute deviation values of the patient distribution shown in panel B. It is clear from the ROC area under the curves (AUC) and their confidence intervals that both tests largely agree. Finally, we plotted all the ROC AUCs for each qPCR threshold (Figure 2D). This shows perfect agreement (AUC = 1) up to Ct 26 and only above Ct 30, a noticeable drop-off to an AUC of 0.95, suggesting that from here on, both diagnostic tests start to disagree slightly. Still, it is important to take the patient population distribution into account when interpreting these thresholds in the higher Ct region.

Next, we calculated multivariate ROC curve analysis based on a linear support vector machine using all five peptides. Classes were defined based on RT-qPCR diagnosis together with log summed MRM area values of the investigated peptide features. Figure S5B,C shows the contribution of the different genes (RT-qPCR) and peptides (MS) to diagnosis as expressed in selected frequency % (SF%). A *t*-test was used to coordinately assess the significance of the difference of each of these measurements between positive and negative patients defined by the other test.

For MS, a clear distinction was seen in SF% between AYN (SF% = 1.0; *p* = 1.1 × 10^–51^) and ADE (SF% = 1.0; *p* = 1.3 × 10^–37^) on the one hand and the three other peptides on the other, i.e., KQQ (SF% = 0.5; *p* = 1.4 × 10^–30^), DGI (SF% = 0.3; *p* = 8.5 × 10^–28^), and NPA (SF% = 0.25; *p* = 2.8 × 10^–26^). Note that all peptides have a low *t*-test statistic and thus do differ significantly between positive and negative patients, yet peptides ADE and AYN again performed best. For the qPCR, the order of performance was E-gene (SF% = 0.9; *p* = 4.2 × 10^–31^), RdRp (SF% =0.7; *p* = 1.4 × 10^–31^), and then N gene (SF% =0.35; *p* = 1.0 × 10^–31^) for the best classifying patients diagnosed by MS. Notably, the E-gene was recently proposed to correlate best to infectivity.^31^ Whether this correlation also implies that MS correlates well with infectivity remains to be determined.

Figure S5D shows the linear correlation between LogInt AYN and Ct separately for the different media in the sample batch, illustrating how the transport medium has only minimal effect after peptide enrichment. Importantly, the initial Cov-MS assay without matrix removal lost linearity around Ct 20–21 for UTM samples with considerable MRM signal interference.^2^ As Cov^2^MS can now measure positive patients past Ct 30 in UTM, this implies an improvement of 10 Ct values, or a 1000-fold (2^10^) increase in assay sensitivity for patient samples in the UTM matrix. Importantly, this patient data also very strongly resembles the results obtained by Hober et al., showing effective inter-laboratory roll-out.^14^

### Detecting Variants of Concern

**Figure 3:**
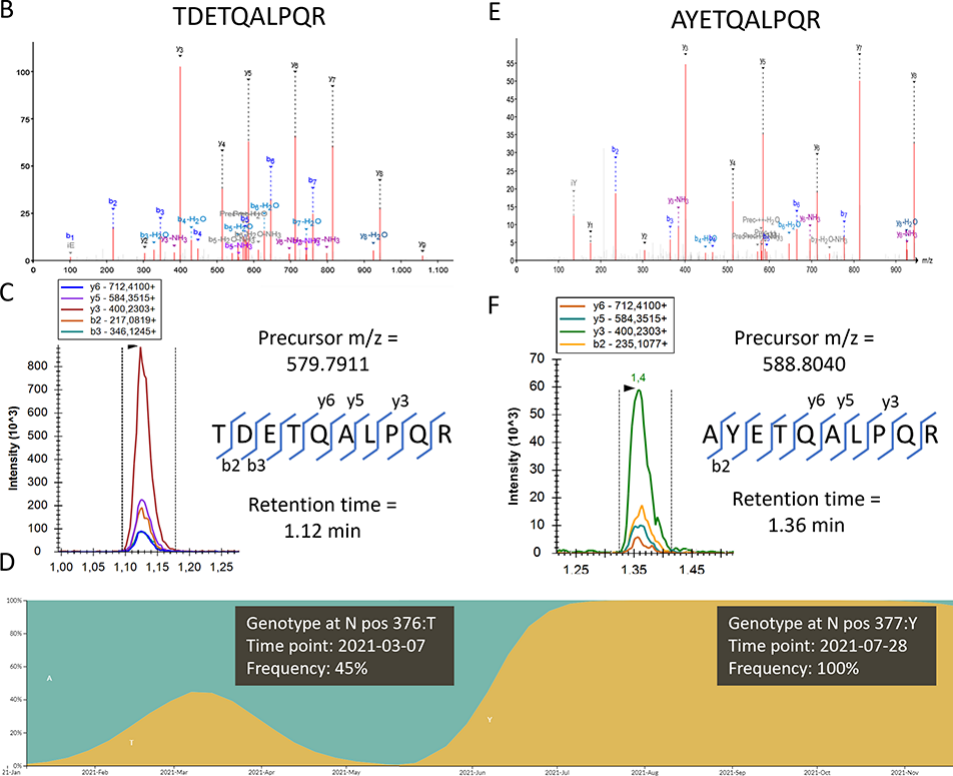
Variant screening. (A) When a patient sample with a missing signal for the ADET peptide was acquired in discovery DDA, a fragment spectrum was found that could be annotated as TDETQALPQR (A376T). (B) The same sample was then reacquired in MRM, this time targeting the mutated peptide by precursor mass and by two b-ions that contain the mutation; a clear signal could be picked up. Adding these to the assay now allows detection of the mutation in all patients in the batch. (C) By checking the GISAID database,^33^ the frequency of this mutation in Belgium showed that this variant was circulating around the time the samples were taken. Not long after, the Delta variant, which contains the (D377Y) mutation, completely replaced the other variants. The figure is composed of two consecutive screenshots. (D) Therefore, a similar approach was applied to specifically identify a biomarker peptide for the Delta VoC and the resulting high resolution MSMS spectrum is shown. (E) This D377Y mutation was still immuno-enriched by the SISCAPA antibody reagent (somewhat less efficiently), and again the target can easily be added to the MRM, albeit at a slightly shifted retention time.

When evaluated using combined peptides ADE + AYN, several patients were significant outliers from the linear correlation with the Ct value (Figure S6). Yet, they behaved more coherently when only the AYN LogInt signal was plotted. Upon detailed inspection of the most positive sample (Ct 11), there was no signal for the ADE peptide, while the heavy standard peptide from the QconCAT was measured as expected (Figure S6, insets). This indicates that the loss in signal cannot be attributed to sample preparation issues.^2^ Since SAT developed anti-peptide antibodies against peptide sequences from genetically stable regions, variant screening is hampered. Still, in a multiplexed assay, the absence of signal from one specific peptide, while maintaining the heavy signal as well as the other peptides, could suggest a mutation in the target sequence. The loss of signal can be attributed to two complementary phenomena: (i) the MRM assay is not measuring the correct transitions at the altered retention time, irrespective of (ii) whether or not the peptide is still captured by the antibody reagent. To assess the latter, the enriched Ct 11 patient sample highlighted in Figure S6 was reanalyzed using discovery data-dependent acquisition (DDA) on a high-resolution TripleTOF 6600+ System (SCIEX, Concord, ON, Canada). Manual inspection of the data showed that the targeted peptide was still present in the sample (it was captured by the antibody), yet the N-terminal alanine (A) in the peptide stretch was mutated to a threonine (T) (A376T) (Figure 3A).

Note that not a single peptide other than the SISCAPA targets could be identified in these samples using DDA, illustrating the selectivity of the method and thus the purity of the Cov^2^MS peptide samples presented for LC–MS analysis. As the peptide was still immuno-purified, a simple switch of acquisition parameters was sufficient to detect it beyond any doubt using MRM upon reinjection (Figure 3B). To verify the validity of this mutation, we tracked the GISAID (https://www.gisaid.org/) database and found that this particular variant was briefly circulating in Belgium around the time of sample collection (Figure 3C). Note that if the peptide had not been enriched by the SISCAPA workflow, the MRM assay would only be capable of detecting the absence of signal. In turn, however, this inspired us to verify if we could enrich and detect the neighboring T377Y mutation known from the Delta B.1.617.2 variant, which arose in Belgium not long after. Indeed, this peptide too could be identified using high-resolution MS (Figure 3D) and was detected by a small adaptation in the transitions of the MRM analysis (Figure 3E), as also seen by others.^34^ The mutation had also induced a small retention time shift.

In conclusion, multiplexing and use of a stable isotope-labeled internal standard together allow the detection of the absence of signal for a single peptide, indicative of a mutation. If the peptide is still sufficiently enriched by the antibody, then it can be readily detected by means of MRM analysis using alternative transition and retention time parameters within the same sample upon the next injection. This emphasizes the importance of always targeting a minimum of at least two peptides in order to avoid positive patients evading detection.

### Peptide Immune-Affinity Enrichment Enables Efficient Sample Pooling

Because of the strongly fluctuating positivity rates of testing throughout a pandemic, it has been proposed to use patient sample pooling to increase throughput and reduce reagent usage for RT-qPCR.^35^ Up to 1/32 pools can be theoretically beneficial for RT-qPCR, depending on the positivity rate of the pandemic. However, for RT-qPCR, this leads to loss in sensitivity since every dilution step reduces the detection by one Ct value, meaning that for a 1/32 dilution experiment, five Ct values in sensitivity are sacrificed and a single positive patient of, e.g., Ct 30 might remain unnoticed in such pooled samples. In contrast, in this second-generation Cov^2^MS assay, the tryptic peptide biomarkers are enriched through antibody binding on magnetic beads. This essentially means that the peptides are extracted from the buffer, theoretically making the assay insensitive to dilution and thus pooling.^13^ Note that we opted to digest the patient samples first and pool fractions of these at the peptide level as a positive pool always requires reanalysis of the separate patients to pinpoint the positives.

**Figure 4:**
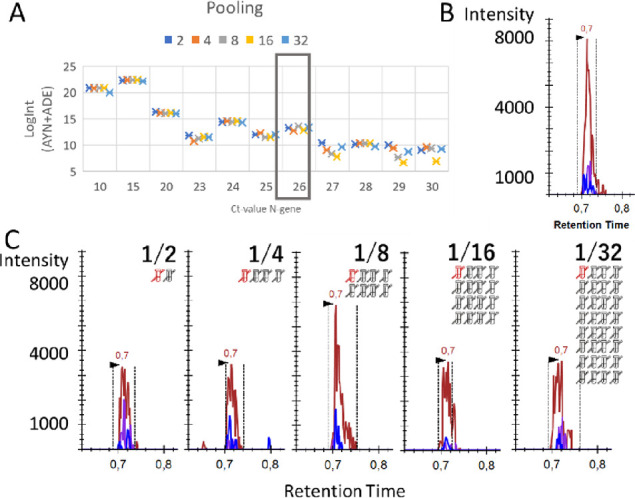
SISCAPA peptide immuno-affinity enrichment is insensitive to patient pooling. (A) 10 μL out of 50 μL of the peptide digest of positive patient samples with different Ct values were diluted with q-PCR-confirmed negative patient samples in five different ratios (1/2, 1/4, 1/8, 1/16, and 1/32), with each dilution corresponding to a loss of one Ct value (*n* = 1). However, by using SISCAPA peptide immuno-affinity enrichment, LC–MS is insensitive to the dilution effect; hence, a similar signal intensity is achieved for each dilution. (B) One positive (red) patient with Ct 26 (boxed in (A)) was manually inspected. The initial measurement of 50 μL sample resulted in a signal for AYN of 7000. (C) When another 50 μL of digest from this patient was spilt into five and diluted 1/2, 1/4, 1/8, 1/16, and 1/32 with a digest of mixture of negative patients, the signal did not decline accordingly, effectively showing how the SISCAPA workflow is insensitive to pooling.

Indeed, for all patients tested, the summed LogInt of ADE + AYN remained stable irrespective of the dilution, i.e., 1/2, 1/4, 1/8, 1/16, and 1/32 dilution with a mixture of negative patient samples (Figure 4A). Figure 4B depicts the raw signal of a patient with Ct 26. Note that only one-fifth of each patient sample (10 μL out of 50 μL) was used for every dilution to create the five different dilutions from a single sample. In a true world setting, a higher amount of sample, e.g., 40 μL, equivalent to using a patient with two Ct higher RT-qPCR results, could be used. For that reason, the raw data from all five dilutions in Figure 4C shows a lower signal than the undiluted sample, yet the signal does not decrease with dilution. This effectively proves that peptide immuno-affinity enrichment is insensitive to pooling, with the starting volume of maximally 1 mL as a limiting factor based on other existing SISCAPA assays. Apart from epidemiological population-wide monitoring as an early warning system, this could also be used to, e.g., screen all passengers of an airplane in a single analysis prior to departure.

### Multiplexing the Assay to Other Viruses

Because of the urgency, most of the previous and current work centered around detecting SARS-CoV-2 in patient samples. However, the approach used can be expanded to include a wider variety of infectious pathogens since an LC–MS instrumental setup simply measures two key properties of an analyte: its hydrophobicity (through its retention time) and its (fragment) masses (through *m*/*z* or MRM detection). This provides manifold higher multiplexing capabilities compared to colorimetric detection techniques. Therefore, including other pathogens into a multiplexed array can be done rapidly and without compromise, with the gradient time and MS duty cycle being the only limiting factors. Still, it can be estimated that a 2 min gradient can potentially harbor enough transitions to monitor up to a dozen pathogens in a single run. We suggest targeting virus nucleoproteins as a first approach because they interact with the virus nucleic acid (providing the linear correlation with RT-qPCR) and are likely to be structurally highly constrained and thus less likely to accumulate mutations. In addition, mutated forms of nucleoproteins are not likely to be selected for because they are less exposed to the immune system of the host, although antigenic drift on viral nucleoproteins due to recognition by cytotoxic T cells should be considered for certain pathogens.^36^ Our peptide target selection (according to the principles described in ref (2)) is depicted in Figure S7.

## Conclusions and Future Perspectives

We have demonstrated that SISCAPA in combination with LC–MS can be used for the high-throughput detection of SARS-CoV-2 peptides in patient samples transported in most commonly used media, as well as in saliva and plasma, down to a limit of detection comparable to a viral load of Ct 31 on the E-gene, which is the most recent estimate for live virus and thus transmissibility.^1^ RT-qPCR is perfectly suited for detecting low or high viral load at incredible sensitivities and throughput. Therefore, if the outcome is that patients are quarantined upon any viral detection to avoid further spread of the virus, then an accurate quantitative measurement of viral load is redundant. Problematically however, based on RT-qPCR results, patients can remain positive long after the infectivity period and in these cases, the need for a sensitive antigen test to accurately determine viral load is paramount.^32^ Additionally, quantitative accuracy will become considerably more important when detecting viral peptides in plasma for prognostic diagnosis, disease state, or treatment outcome.

## Supporting Information Available

Additional experimental details on the viral cultures, RT-qPCR, dilution series, precision experiments, sample pooling, and an extended description of the used LC–MS methods (PDF)
