# Supplementary material for: Cov2MS: An Automated and Quantitative Matrix-Independent Assay for Mass Spectrometric Measurement of SARS-CoV-2 Nucleocapsid Protein
Source: Anal Chem. 2022 Dec 9;94(50):17379–87. doi: 10.1021/acs.analchem.2c01610 (PMC9773173; doi:10.1021/acs.analchem.2c01610)
Supplement: Supplementary file 1 — ac2c01610_si_001.pdf [file ac2c01610_si_001.pdf]

Supporting Information
Cov²MS: an automated, and quantitative matrix-independent assay for mass spectrometric measurement of SARS-CoV-2 nucleocapsid protein.

Bart Van Puyvelde [1], Katleen Van Uytfanghe [2], Laurence Van Oudenhove [3], Ralf Gabriels [4,5], Tessa Van Royen [4,6], Arne Matthys [4,6], Morteza Razavi [7], Richard Yip [7], Terry Pearson [7], Nicolas Drouin [8], Jan Claereboudt [3], Dominic Foley [9], Robert Wardle [9], Kevin Wyndham [9], Thomas Hankemeier [8], Donald Jones [10,11], Xavier Saelens [4,6], Geert Martens [12], Christophe P. Stove [2], Dieter Deforce [1], Lennart Martens [4,5], Johannes P.C. Vissers [9], N. Leigh Anderson [7], Maarten Dhaenens [1*]

AUTHOR ADDRESS

[1] ProGenTomics, Laboratory of Pharmaceutical Biotechnology, Ghent University, 9000 Ghent, Belgium

[2] Laboratory of Toxicology, Department of Bioanalysis, Faculty of Pharmaceutical Sciences, Ghent University, 9000 Ghent, Belgium

[3] Waters Corporation, 2600 Antwerp, Belgium

[4] VIB-UGent Center for Medical Biotechnology, VIB, 9000 Ghent, Belgium

[5] Department of Biomolecular Medicine, Ghent University, 9000 Ghent, Belgium

[6] Department of Biochemistry and Microbiology, Ghent University, Ghent, Belgium

[7] SISCAPA Assay Technologies, Inc., Box 53309, Washington, DC 20009, United States of America and Victoria, BC Canada

[8] Division of Systems Biomedicine and Pharmacology, Leiden Academic Centre for Drug Research, Leiden University, 2333 AL Leiden, The Netherlands

[9] Waters Corporation, Wilmslow SK9 4AX, United Kingdom and Milford, MA 01757, United States of America

[10] Leicester Cancer Research Centre, RKCSB, University of Leicester, UK and John and Lucille van Geest biomarker facility, Cardiovascular Research Centre, Glenfield Hospital, Leicester LE1 7RH, United Kingdom

[11] The Department of Chemical Pathology and Metabolic Diseases, Level 4, Sandringham Building, Leicester Royal Infirmary, Leicester LE1 7RH, United Kingdom

[12] AZ Delta Medical Laboratories, AZ Delta General Hospital, 8800 Roeselare, Belgium

*Corresponding author: maarten.dhaenens@ugent.be

ABSTRACT: The pandemic readiness toolbox needs to be extended, targeting different biomolecules, using orthogonal experimental set-ups. Here, we build on our Cov-MS effort using LC-MS, adding SISCAPA technology to enrich proteotypic peptides of the SARS-CoV-2 nucleocapsid (N) protein from trypsin-digested patient samples. The Cov²MS assay is compatible with most matrices including nasopharyngeal swabs, saliva, and plasma and has increased sensitivity into the attomole range, a 1000-fold improvement compared to direct detection in matrix. A strong positive correlation was observed with qPCR detection beyond a quantification cycle of 30-31, the level where no live virus can be cultured. The automatable sample preparation and reduced LC dependency allow analysis of up to 500 samples per day per instrument. Importantly, peptide enrichment allows detection of the N protein in pooled samples without sensitivity loss. Easily multiplexed, we detect variants and propose targets for Influenza A and B detection. Thus, the Cov²MS assay can be adapted to test for many different pathogens in pooled samples, providing longitudinal epidemiological monitoring of large numbers of pathogens within a population as an early warning system.

Table of contents

Supplementary Methods S-2

Supplementary Figures and Tables S-3

Figure S1. .... S-3
Table S1 .... S-4
Figure S2. .... S-5
Figure S3 .... S-5
Figure S4. .... S-6
Figure S5 .... S-6
Figure S6 .... S-7
Figure S7 .... S-7
References S-8

Supplementary Methods

Virus cultures. MDCK (Madin–Darby canine kidney) cells were cultured and seeded in complete DMEM medium containing fetal bovine serum (FBS) and non-essential amino acids. The cells were seeded for confluency in a 225 ml Falcon flask at 37°C and 5% CO2. Upon reaching confluency, the medium was refreshed with serum-free DMEM containing 4 µg/ml of N-p-Tosyl-L-phenylalanine chloromethyl ketone (TPCK) and the adherent cells were infected with mouse-adapted H3N2 (X47) or mouse-adapted H1N1/2009 Pandemic influenza A virus. One day post infection, cytopathic effects were observed and the supernatant was harvested. The supernatant was centrifuged for 15 mins (4° C; 300 x g) to pellet the cell debris and the clarified supernatant was pooled and frozen at -80°C. A hemagglutination assay was performed which revealed a titre of 29 and 24, respectively, for the X47 and H1N1/2009 pandemic virus-containing supernatant. For this assay, a 2-fold dilution series of the supernatant was made in PBS in a U-bottomed 96 well plate. PBS was used as a negative control. Fifty µl of 1% turkey RBC were added to 50 µl diluted supernatant sample and the mixture was incubated for 1 hr at room temperature, after which RBC agglutination was visually determined.

MDCK cells were cultured, seeded and infected according to the protocol outlined above. Cells were infected with B/Memphis/12/97, B/Washington/02/2019, or B/Phuket/3073/2013 and the cell culture supernatant was harvested and clarified as above, at 3 days post infection, when cytopathic effect was evident. Upon harvest, a hemagglutination assays were performed to confirm viral proliferation. Hemagglutination titers of 212, 28 and 27, respectively, were obtained for the B/Memphis/12/97, B/Washington/02/2019 and B/Phuket/3073/2013 virus preparations.

RT-qPCR protocol. RNA was extracted from nasopharyngeal swabs using a STARMag 96 x 4 Viral DNA/RNA 200 C Kit (Seegene Technologies, Walnut Creek, CA, USA) on the Hamilton STARlet workstation, followed by real-time PCR using the Allplex SARS-CoV-2 assay. PCR amplification was performed using a CFX96 real-time thermal cycler (Bio-Rad Laboratories) and data were analysed with the SARS-CoV-2 Viewer (Seegene). The presence or absence of SARS-CoV-2 RNA was determined by RT-PCR combined with multiplexed fluorescent probing, in which three different SARS-CoV-2 genes i.e. E-gen (FAM), RdRP (Cal Red 610) and N gene (Quasar 670) and an internal control (HEX) were targeted.

LC gradient. For the 1-minute run, gradient elution was performed at 1 mL/min with initial inlet conditions at 7 % B, increasing to 40 % B over 0.3 min, followed by a column wash at 90 % B for 0.25 min and a return to initial conditions at 7 % B. The total run time was 0.8 min. For the 2-minute min run, gradient elution was performed at 0.8 mL/min with initial inlet conditions at 5 % B, increasing to 15 % B from 0.15 to 0.35 min and at a steady state for 0.25 min. Subsequently, over 0.4 min, gradient B was increased to 25%, followed by a column wash at 90 % B for 0.25 min and returning to initial conditions at 5 % B. The total run time was 1.8 min. For the 8-minute run, gradient elution was performed at 0.6 mL/min with initial inlet conditions at 5 % B, increasing to 33 % B over 5.5 min, followed by a column wash at 90 % B for 1.4 min and a return to initial conditions at 5 % B. The total run time was 8 min. The Xevo TQ-XS conditions were as follows: capillary voltage 0.5 kV, source temperature 150° C, desolvation temperature 600° C, cone gas flow 150 L/h, and desolvation gas flow 1000 L/h. The MS was calibrated at unit mass resolution for MS1 and MS2. Endogenous and the corresponding stable isotope labelled peptides were detected using MRM acquisition with experimental details described in **Table S1**.

Dilution series. A serial dilution was performed using the SARS-CoV-2 negative nasopharyngeal swab pools to obtain a dilution series with the following concentrations of N: 10000, 2000, 400, 80, 16, 4, 2 and 0 amol/µL. Since each

dilution starts from 180 µL of sample, the theoretical amount of sample loaded on column is calculated as following: =(180 µL* X amol/µL)/(50 µL)*10 µL. For example a 2 amol/µL sample corresponds to 72 amol NCAP on column.

Intra- and inter-day precision was determined by Waters Corporation using the SARS-CoV-2 RUO kit [1]. In short, 20 µl of denaturant mixture (1 % (w/v) RapiGest [Waters Corporation, Milford, MA]) in 500 mM ammonium bicarbonate were aliquoted into a 96-well plate. Next, 180 µl of the NCAP dilutions were pipetted into the same plate, followed by a shaking step of 30 seconds at 1200 rpm and an incubation step of 15 min at 56°C. After cooling the plate for 5 minutes, 20 µl trypsin solution (5 mg/ml trypsin in 10 mM HCl) was added. After mixing at 1200 rpm for 30 s, the samples were digested at 37°C for 30 min and thereafter quenched by addition of 20 µL trypsin stopping agent (0.5 mg/ml of Tosyl-L-lysyl-chloromethane hydrochloride in 10 mM HCl). The sample plate was mixed at 1200 rpm for 5 minutes, before adding 20 µL of SIL peptide mixture solution and mixing again on a shaker at 1200 rpm for 30 s. Finally, SISCAPA peptide enrichment was performed by adding 10 µL of ADETQALPQR and AYNVTQAFGR antibodies.

Sample pooling. A dozen of nasopharyngeal patient samples screened negative for SARS-CoV-2 using RT-qPCR (Ct > 40) were pooled to create five different dilutions, i.e. ½, ¼, 1/8, 1/16 and 1/32, starting from confirmed positive patient samples with diverse viral loads (Ct 10, 15, 20, 23, 24, 25, 26, 27, 28, 29 and 30). To 180 µL of undiluted sample seven volumes of ice-cold acetone were added, followed by a centrifugation step of 10 minutes at 16,000g and 0°C. After removing the supernatant, 50 µL of trypsin digestion buffer was added before the sample was incubated at 37°C for 30 minutes. Afterwards, the sample was divided in five equal fractions of 10 µL and to each of the fractions a different amount of volume of negative digested patient samples were added (i.e. 10, 30, 70, 150 and 310 µL). Each dilution was then subjected to the SISCAPA peptide immuno-enrichment protocol using a similar amount of beads. The final elution for all samples was performed in 50µL of 1% FA.

Supplementary Figures and Tables

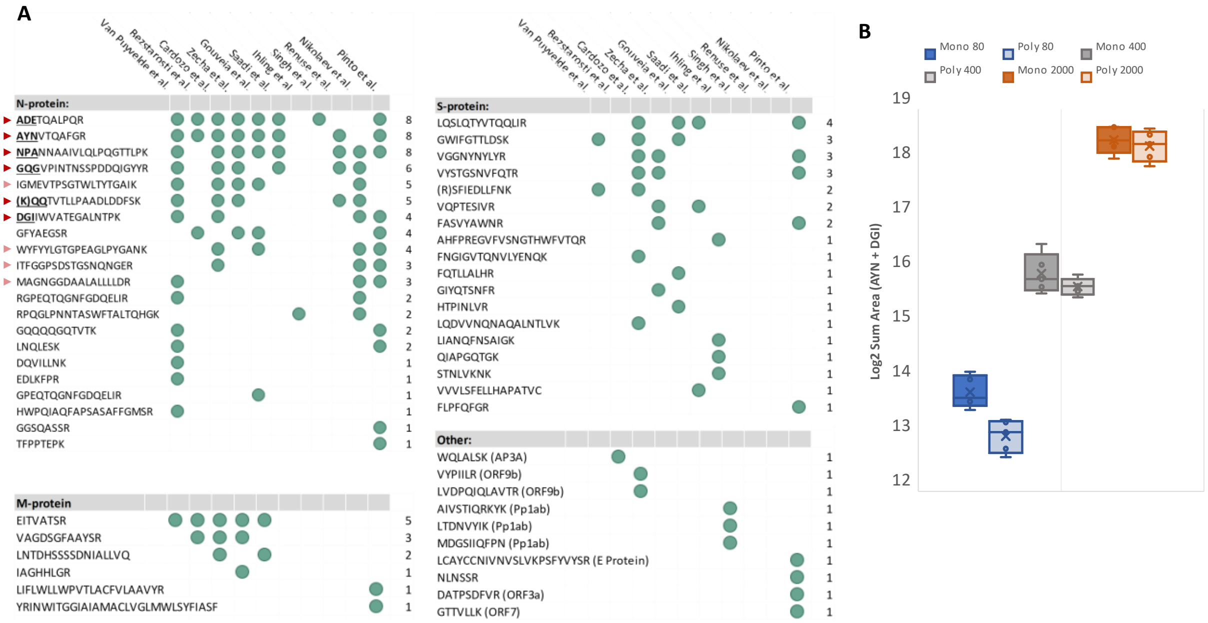

Figure S1. A) SARS-CoV-2 peptides detected in early MS-based efforts. Red arrowheads indicate peptides targeted for SISCAPA antibody-based peptide enrichment (opaque: polyclonal only). The bold underlined initial three letters depict the peptide abbreviations used throughout the manuscript. **B)** Intensity comparison (LogSum area under the curve) between polyclonal and monoclonal antibodies against two target peptides (AYN and DGI) spiked in three different concentrations (80-400-2000 amol/µL) in Bioer UTM background.

Table S1. MRM parameters for the six SISCAPA target peptides. Transitions highlighted in orange are used as stable isotope labelled standard.

Peptide	MRM	Cone Voltage (V)	Collision Energy (V)	Retention time (1min)	Retention time (2min)	Retention time (8min)	Scan window (1min)	Scan window (2min)	Scan window (8min)
ADETQALPQR	564.8 > 712.4 (y6)	35	24		0.48	1.13		0.1-0.7	0.5-1.6
	564.8 > 584.4 (y5)	35	20		0.48	1.13		0.1-0.7	0.5-1.6
	564.8 > 400.2 (y3)	35	17		0.48	1.13		0.1-0.7	0.5-1.6
	**572.3 > 407.2 (y3)**	35	17		0.48	1.13		0.1-0.7	0.5-1.6
AYNVTQAFGR	563.8 > 892.5 (y8)	35	17	0.4	0.72	2.43	0.15-0.8	0.5-0.9	1.9-2.9
	563.8 > 778.4 (y7)	35	17	0.4	0.72	2.43	0.15-0.8	0.5-0.9	1.9-2.9
	563.8 > 679.4 (y6)	35	20	0.4	0.72	2.43	0.15-0.8	0.5-0.9	1.9-2.9
	563.8 > 349.2 (b3)	35	17	0.4	0.72	2.43	0.15-0.8	0.5-0.9	1.9-2.9
	**571.3 > 689.3 (y6)**	35	20	0.4	0.72	2.43	0.15-0.8	0.5-0.9	1.9-2.9
GQGVPINTNSSPDDQIGYYR	727.7 > 1126.5 (y9)	35	23		0.81	2.62		0.6-1	2.1-3.1
	727.7 > 558.3 (y4)	35	23		0.81	2.62		0.6-1	2.1-3.1
	727.7 > 563.8 (y9++)	35	23		0.81	2.62		0.6-1	2.1-3.1
	727.7 > 342.2 (b4)	35	23		0.81	2.62		0.6-1	2.1-3.1
	**736.7 > 570.2 (y9++)**	35	23		0.81	2.62		0.6-1	2.1-3.1
KQQTVTLLPAADLDDFSK	664.0 > 1078.5 (y10)	35	18	0.44	1.18	3.8	0.15-0.8	1-1.4	3.3-4.3
	664.0 > 539.8 (y10++)	35	14	0.44	1.18	3.8	0.15-0.8	1-1.4	3.3-4.3
	664.0 > 799.5 (b7)	35	22	0.44	1.18	3.8	0.15-0.8	1-1.4	3.3-4.3
	**671.3 > 545.2 (y10++)**	35	14	0.44	1.18	3.8	0.15-0.8	1-1.4	3.3-4.3
NPANNAAIVLQLPQGTTLPK	687.4 > 841.5 (y8)	35	17		1.19	3.9		1-1.4	3.4-4.4
	687.4 > 766.4 (b8)	35	23		1.19	3.9		1-1.4	3.4-4.4
	687.4 > 433.2 (b9)	35	20		1.19	3.9		1-1.4	3.4-4.4
	**696.0 > 851.4 (y8)**	35	17		1.19	3.9		1-1.4	3.4-4.4
DGIIWVATEGALNTPK	842.9 > 286.1 (b3)	35	30		1.24	4.15		1-1.7	3.7-5
	562.3 > 700.4 (y7)	35	10		1.24	4.15		1-1.7	3.7-5
	562.3 > 700.4 (y13++)	35	18		1.24	4.15		1-1.7	3.7-5
	**852.4 > 289.1 (b3)**	35	30		1.24	4.15		1-1.7	3.7-5

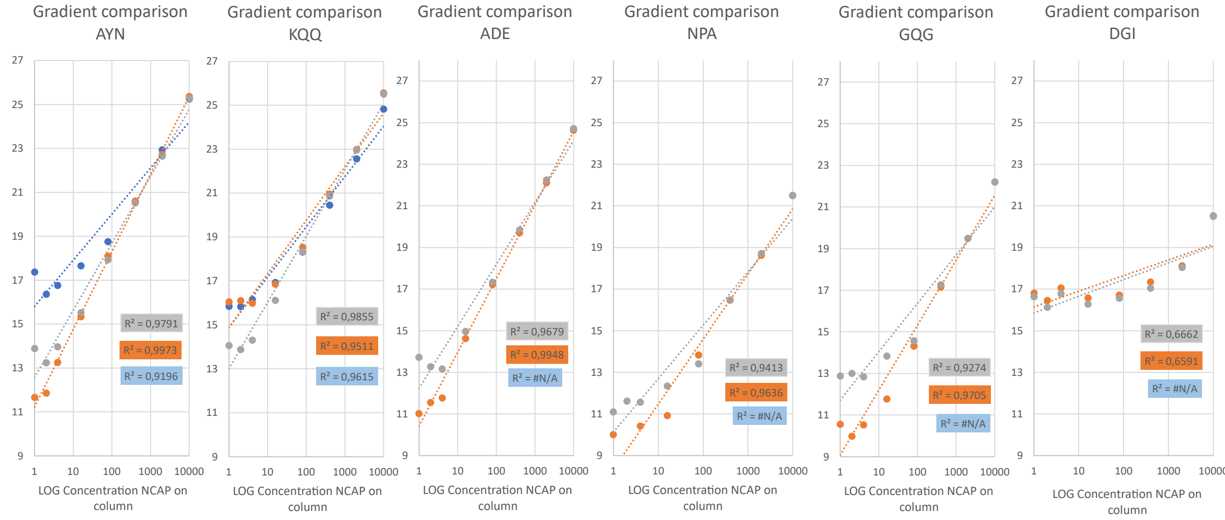

Figure S2. Response of six SISCAPA peptide targets (AYNVTQAFGR, KQQTVTLLPAAD-LDDFSK, ADETQALPQR, NPANNAAIVLQLPQGTTLPK, GQGVPINTNSSPDDQIGYYR and DGIIWVATEGALNTPK) immunopurified from a NCAP_SARS2 dilution series and analysed using three different LC gradients (Blue: 1 min, Orange: 2 min and Grey: 8 min) on the Xevo TQ-XS. Here, the x- and y-axis represent the LogConcentration of Nucleoprotein and the corresponding summed peptide Log2Int of the MS signal extracted from Skyline, respectively. Overall, a 2-minute gradient demonstrated the highest linearity, with the ADE and AYN peptides performing best in terms of MRM sensitivity, especially in the low intensity range close to the lower limit of quantification. While the KQQ peptide showed the best linearity using the 8-minute LC gradient, manual inspection of the data showed that the apparent higher signal within the low intensity range for the shorter gradients was caused by carry-over in-between dilution series.

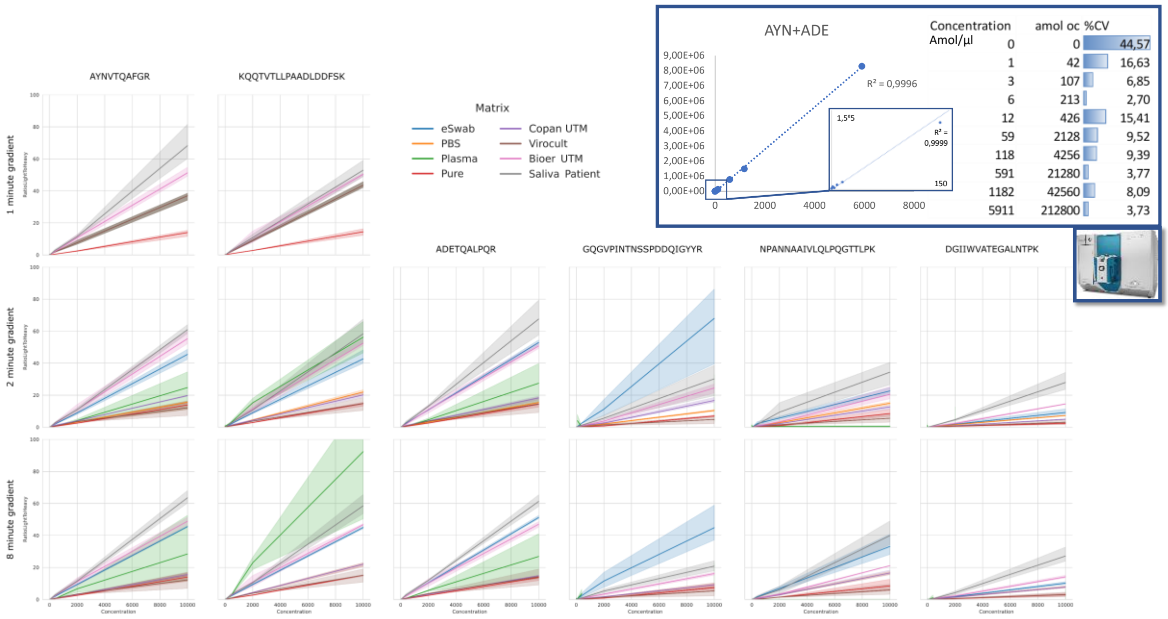

Figure S3. Light/heavy ratio of six SISCAPA peptides in eight different matrices (i.e. eSwab, PBS, Copan UTM, Virocult, Bioer UTM, Plasma, 100 mM NH_4HCO_3 and Saliva Patient) using three different gradients. Inset demonstrates the inter-lab reproducibility of the method as a similar LOQ is achieved when an almost similar dilution series in Bioer UTM is measured on a SCIEX 7500 Triple Quad system at Leiden University.

A

%CV

amol oc	Pure	PBS	eSwab	Virocult	UTM	Bioer_UTM	SynSaliva	Saliva_Patient	Plasma
0	36,26	50,91	44,58	37,49	33,21	42,18	82,31	37,77	66,60
72	62,50	45,05	14,13	27,98	14,57	23,77	104,95	22,70	49,70
144	33,42	11,60	33,38	15,12	6,08	15,37	97,45	13,06	11,12
576	15,15	21,47	7,57	12,11	13,06	6,14	93,06	21,49	31,53
2880	4,38	13,25	6,52	5,94	5,59	0,72	49,95	10,38	59,17
14400	5,30	13,10	1,93	14,75	21,93	32,06	70,73	19,61	53,26
72000	12,47	3,89	21,72	12,70	1,78	5,75	70,20	19,45	36,57
360000	28,49	15,66	9,08	11,28	4,66	2,32	45,54	11,91	41,75

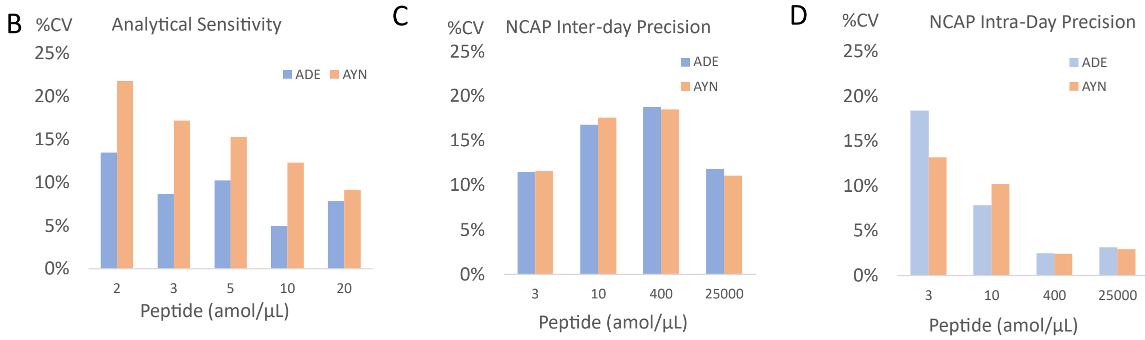

Figure S4. **(A)** Percent coefficient of variation (%CV) of recombinant N protein spiked in a dilution series in different nasopharyngeal swab matrices (n=3): 100 mM NH_4HCO_3 (referred to as “Pure”), PBS, Copan eSwab, Sigma Virocult, Copan UTM, Bioer UTM, Synthetic saliva, Saliva patient and Plasma. **(B)** Analytical sensitivity of the target peptides AYNVTQAFGR (AYN) and ADETQALPQR (ADE). The sensitivity assessment meets the acceptance criteria, with precision at an LoQ of 3 amol/µL ≤17.2% and bias within ±19.8%. **(C&D)** Intra- and inter-day precision analysis on the two main target peptides (ADE and AYN). The five-day precision assessment meets the acceptance criteria, with inter-day and intra-day precision ≤17.4% across five runs.

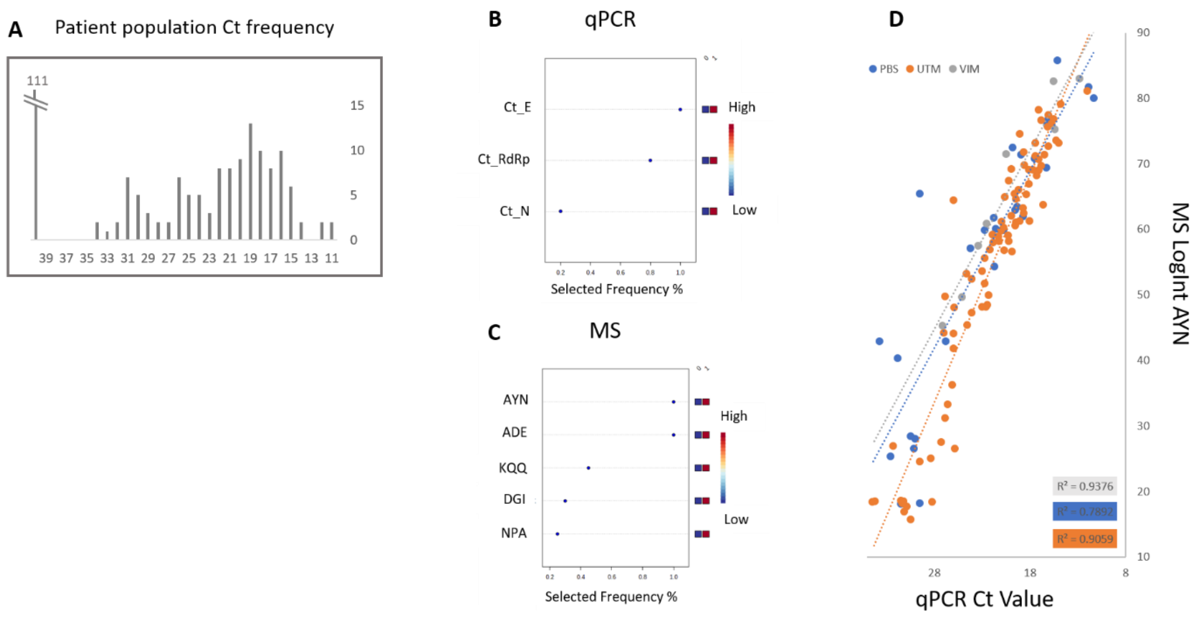

Figure S5. A) The distribution of the patient population over their measured E-gene Ct values. Contribution of the different **B**) genes (qPCR) and **C)** peptides (MS) to diagnosis as expressed in Selected Frequency % (SF%). **D)** Linear correlation between summed MRM LogInt AYN and Ct for the different media (PBS, UTM and VIM) in the sample batch separately.

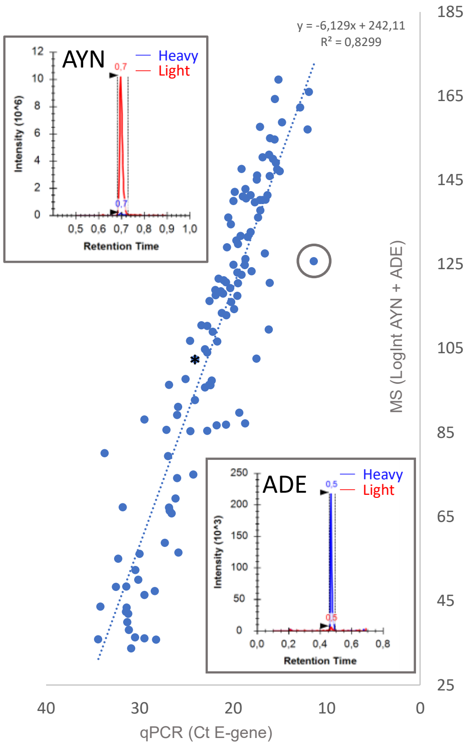

Figure S6. (A) Several patients had a summed ADE + AYN LogInt that was significantly lower than what was expected based on the linear correlation with the Ct value. Manual inspection of one patient (with Ct 11, circled in grey), showed how only the light signal of the ADE peptide and not the one from the AYN peptide had disappeared (insets).

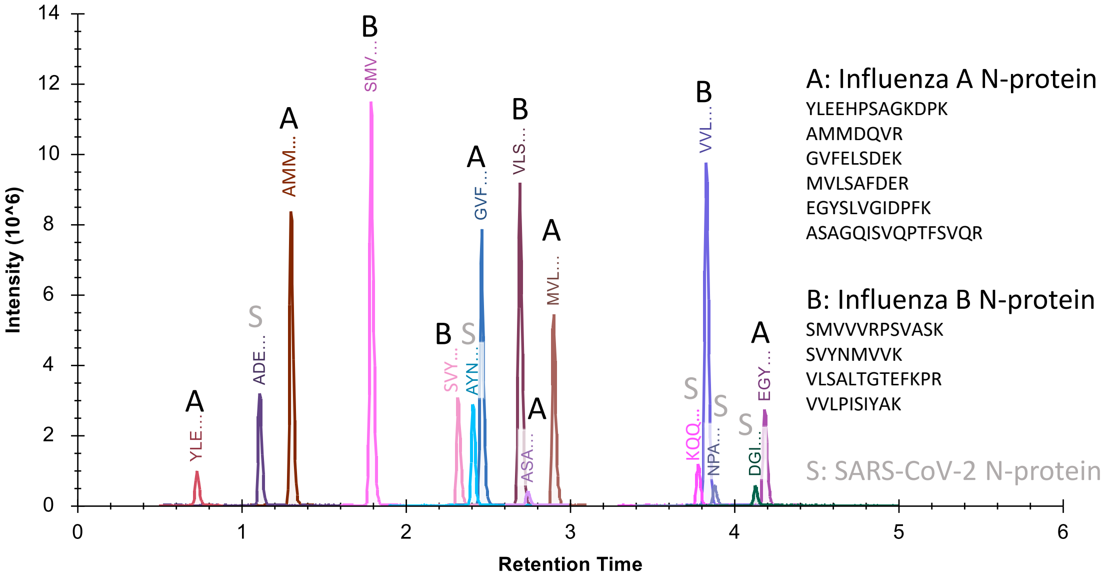

Figure S7. Preliminary data illustrating multiplexed analysis for SARS-CoV-2, Influenza A and Influenza B. The Phase 1 workflow described for the previously published Cov-MS work was applied to viral cultures of different Influenza A (H1N1/2009 and H3N2/X47) and Influenza B (Memphis, Phuket and Washington) strains. Proteotypic peptides specific for both strains were selected and appended to the peptide selection of SARS-CoV-2. Subsequently, an equimolar dilution series of recombinant nucleoproteins from these three viruses was created in NH4HCO3 as a proof of concept. The peptides from both Influenza strains ionized even better than those from SARS-CoV-2, highlighting the potential of SISCAPA-LC-MS for multiplexed testing.

References

(1) Foley, D.; Wardle, R.; Ferries, S.; Pattison, R.; Warren, J.; Calton, L. J. Advancing Research with the SARS-CoV-2 LC-MS Kit (RUO) | Waters https://www.waters.com/webassets/cms/library/docs/720007266en.pdf.
